# Proton‐Radiation Hardening of High‐Speed Ge/Si Integrated Photodetectors for Space Applications

**DOI:** 10.1002/nap2.70165

**Published:** 2026-06-12

**Authors:** Junguo Xu, Yingying Zhong, Xiongping Bao, Junfeng Chen, Wenjie Peng, Haotian Hu, Yiti Xiong, Weiye Xia, Xiao Chen, Meisong Liao, Jianhui Bin, Yuchen Wang, Libing Zhou, Weibiao Chen

**Affiliations:** ^1^ Shanghai Institute of Optics and Fine Mechanics, Chinese Academy of Sciences Shanghai China; ^2^ University of Chinese Academy of Sciences Beijing China

**Keywords:** Ge/Si photodetector, hydrogen loading, proton irradiation, radiation hardening

## Abstract

High‐speed Ge/Si integrated photodetectors are essential for spaceborne photonic links, yet proton irradiation can strongly increase leakage current and noise, compromising long‐term reliability. Here, we performed a broad‐spectrum proton‐irradiation study on high‐speed Ge/Si integrated photodetectors and validated hydrogen loading as a radiation‐hardening approach. An RCF‐calibrated energy–fluence spectrum combined with Geant4 energy‐deposition simulations enabled dose‐calibrated testing at cumulative deposited doses of 158, 316, 790, and 1580krad in the silicon layer under a laser–plasma‐driven broadband proton beam with a maximum energy of 10.75MeV. For untreated devices, the bandwidth decreased from 44.52GHz to 32.09GHz at 1580krad, whereas the dark current at a 3V reverse bias increased from 6.19nA to 8.67× its preirradiation level. Hydrogen‐loaded devices remained functional and exhibited improved tolerance, retaining ∼35GHz bandwidth (78.6% of the preirradiation reference) while limiting the dark‐current increase to 1.9× at 1580krad. This demonstrates a practical hardening route for space‐relevant integrated photodetectors.

## Graphical abstract

Hydrogen loading is demonstrated as a radiation‐hardening strategy for high‐speed Ge/Si integrated photodetectors under broadband proton irradiation. By suppressing irradiation‐induced leakage‐current growth and mitigating bandwidth degradation, hydrogen‐loaded devices maintain high‐speed operation and improved stability, providing a practical route toward radiation‐tolerant silicon‐photonic receivers for spaceborne optical links.

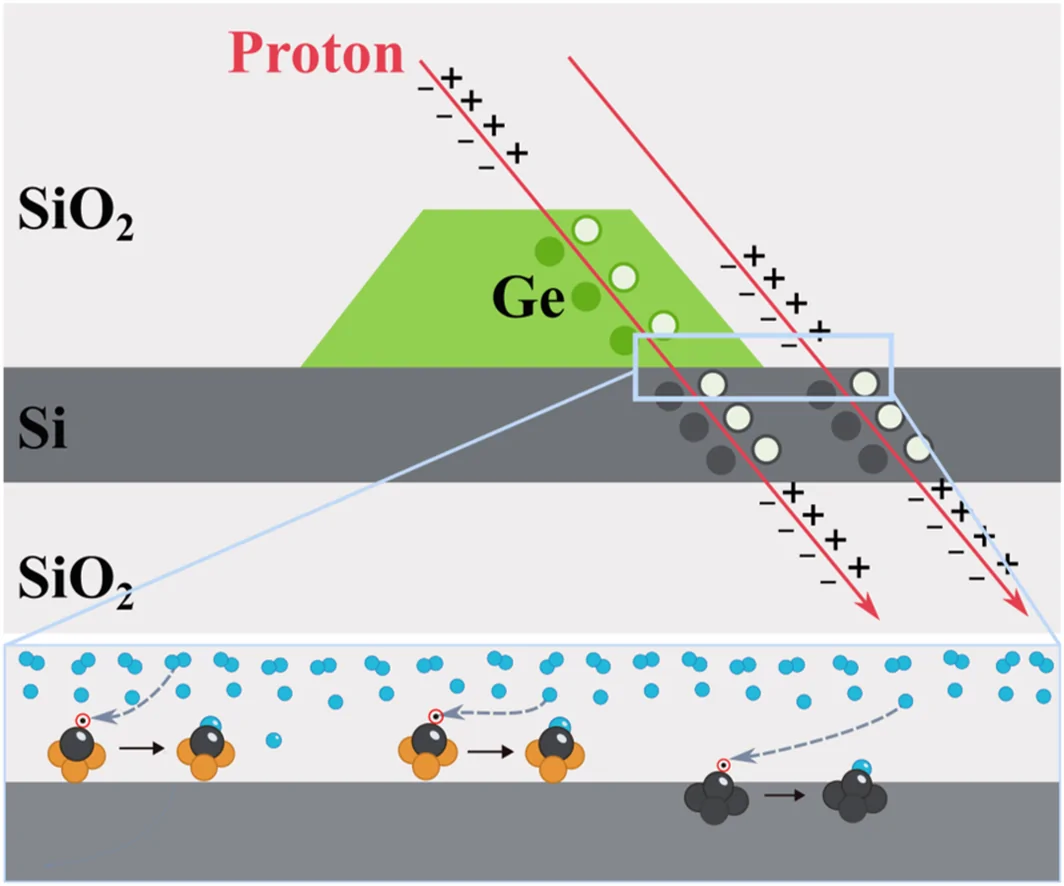

## 1 Introduction

Integrated circuits are currently confronting dual constraints in both physics and economics. Whether it is the staggering energy consumption and heat dissipation resulting from quantum effects or the exponentially rising costs of research, development, and manufacturing, the advancement of integrated circuits has become increasingly challenging. Consequently, photonic integrated circuits (PICs) have emerged as a promising alternative. Compared to traditional electronic integrated circuits, photonic information processing offers advantages such as lower power consumption, significantly higher bandwidth, and intrinsic immunity to electromagnetic interference [1, 2, 3]. Among many promising material platforms for PICs, including Si, Si_3_N_4_, III–V semiconductors, and more [4, 5, 6], silicon photonics has attracted increasing attention from both industry and academia due to its advantages such as low cost, high scalability, low power consumption, and CMOS compatibility [7, 8]. Silicon photonics has now been widely applied in fields such as optical interconnects [9, 10], microwave photonics [11], and artificial intelligence [12], among others.

Simultaneously, silicon photonics has also demonstrated significant potential for space applications. In the context of aerospace, the improvements it offers in terms of stability, system size, weight, power consumption, and cost are even more pronounced than those in terrestrial applications. This makes silicon photonics a promising candidate for space‐based optical communication instruments [13, 14]. To achieve on‐chip optical communication and signal processing, passive devices (e.g., optical waveguides, beam splitters) and active devices (e.g., modulators, photodetectors) play critical roles in signal transmission and manipulation and in signal modulation and detection, respectively. Although silicon‐photonic devices have achieved relative maturity and high reliability in terrestrial applications, their stability and operational lifetime face inevitable threats from the diverse range of space radiation in extraterrestrial environments. The space radiation environment is notably complex, comprising various radiation sources with wide energy and dose distributions. Primary radiation types include electromagnetic radiation (such as X‐rays and gamma rays) originating from solar and galactic cosmic rays, as well as pervasive particle radiation (e.g., high‐energy protons and heavy ions).

Encouragingly, studies have shown that passive devices exhibit relatively low sensitivity to radiation‐induced damage and maintain favorable performance stability under various radiation conditions [15, 16, 17, 18, 19]. In contrast, active devices exhibit relatively high sensitivity to charge‐related damage. The Mach–Zehnder modulator is widely adopted due to its excellent stability and versatility, and consequently, a series of studies has been conducted on its radiation‐induced degradation. High‐energy radiation—such as X‐rays, gamma rays, neutron irradiation, and proton irradiation—can significantly threaten the performance stability and operational lifetime of Mach–Zehnder modulators. It has been shown that heavily doped and shallow‐etched PN junctions can improve the radiation hardness of such devices [20, 21, 22, 23]. Compared to modulators, radiation tolerance studies on photodetectors remain relatively limited. Research indicates that X‐ray and gamma‐ray irradiation can lead to a notable increase in the reverse‐bias dark current of photodetectors, whereas their bandwidth remains largely unaffected [20, 24].

For space‐based information processing systems, the optical field must first be converted into an electrical signal before it can undergo amplification, sampling, demodulation, and digital processing. The performance of the photodetector directly determines key system parameters such as signal‐to‐noise ratio, bandwidth, dynamic range, and linearity, which, in turn, influence the suitability of modulation formats, bit‐error rate, or ranging accuracy, as well as power consumption and thermal design. At the same time, space‐environment factors—including radiation, severe temperature variations, and long‐term on‐orbit reliability—can lead to increased dark current, gain degradation, and responsivity drift. These effects make the photodetector a critical limiting component in achieving the targeted system specifications.

In addition to Si‐ and Ge‐based photodetectors, GaN‐based photodetectors have also attracted extensive attention for space‐related and harsh‐environment sensing, particularly in the ultraviolet spectral range. Benefiting from its wide bandgap, high thermal stability, chemical robustness, and radiation tolerance, GaN has been employed in ultraviolet photodetectors for liquid‐level sensing in harsh environments, omnidirectional UV detection for navigation‐related scenarios, temperature‐compensated UV sensing, and reliable operation under low‐temperature or thermally disturbed conditions [25, 26, 27, 28, 29]. These studies demonstrate the broad potential of GaN‐based photodetectors for robust ultraviolet sensing in environments such as aerospace, nuclear reactors, and other harsh operating conditions.

Silicon possesses an inherently weak optoelectronic response, making it generally unsuitable as an absorbing material for high‐performance photodetectors. Although III–V semiconductors and wide‐bandgap materials such as GaN are important for specific detection bands and harsh‐environment sensing, near‐infrared silicon‐photonic communication links require photodetectors that are compatible with the silicon‐photonic platform and exhibit efficient absorption at communication wavelengths. In this context, germanium has emerged as the preferred choice for silicon‐based photodetection. This preference stems from its compatibility with mature direct epitaxial growth on silicon substrates, coupled with its strong absorption and efficient photoelectric conversion capabilities in the near‐infrared band. As a result, Ge/Si photodetectors are now widely utilized in high‐sensitivity near‐infrared detection applications [30, 31, 32, 33, 34].

In this work, we conducted a systematic proton‐irradiation study on high‐speed Ge/Si integrated photodetectors under space‐relevant irradiation conditions using a laser–plasma‐driven broadband proton beam with a maximum energy of 10.75MeV. Four irradiation levels were employed to evaluate irradiation‐induced performance degradation in a controlled and comparable manner. The dark current at a reverse bias of 3V and the 3‐dB bandwidth were used as key metrics to quantify the static leakage/noise floor and the dynamic response, respectively. To improve radiation tolerance, we introduced a hydrogen preloading process to reduce the electrical activity of irradiation‐related defect states and suppress defect‐assisted leakage pathways. Experimental results show that untreated devices suffer severe degradation. At the highest irradiation level, the dark current increases from 6.19nA to 8.67× its preirradiation value under a 3V reverse bias, whereas the bandwidth decreases from 44.52GHz to 32.09GHz. In contrast, hydrogen‐loaded devices exhibit markedly improved robustness. At the same irradiation level, the dark‐current increase is limited to 1.9×, and the bandwidth is preserved at approximately 35GHz (78.6% of the preirradiation reference). These results verify the effectiveness of hydrogen‐assisted irradiation hardening and support the development of space‐compatible Ge/Si integrated photodetectors.

## 2 Theory and Simulation

### 2.1 Irradiation Damage Mechanism

Radiation‐induced damage in semiconductor devices is generally classified into two primary categories: total ionizing dose (TID) effects and displacement damage dose (DDD) effects. TID effects predominantly occur in the oxide/insulator and at the oxide–semiconductor interface. Ionizing radiation generates electron–hole pairs in the oxide. As illustrated in Figure 1a, electrons, owing to their higher mobility, are readily swept out by the electric field or rapidly recombine, whereas holes, with much lower mobility, are more likely to drift/diffuse within the oxide and become trapped, leading to oxide‐trapped charge and the formation of interface states at the Si/${\text{SiO}}_{2}$ interface (e.g., $P_{b}$ centers). The resulting fixed charges and interface traps modify the local electric field and energy‐band profile, giving rise to band bending and threshold‐voltage shifts and ultimately causing electrical degradation such as increased leakage current and noise as well as reduced carrier mobility. For photodetectors, oxide/interface traps enhance surface recombination and introduce additional generation–recombination pathways, increasing dark current and noise while altering the effective junction field and depletion width. These changes degrade key performance metrics, including responsivity and quantum efficiency, and may further impair bandwidth and linearity [35, 36].

**FIGURE 1:**
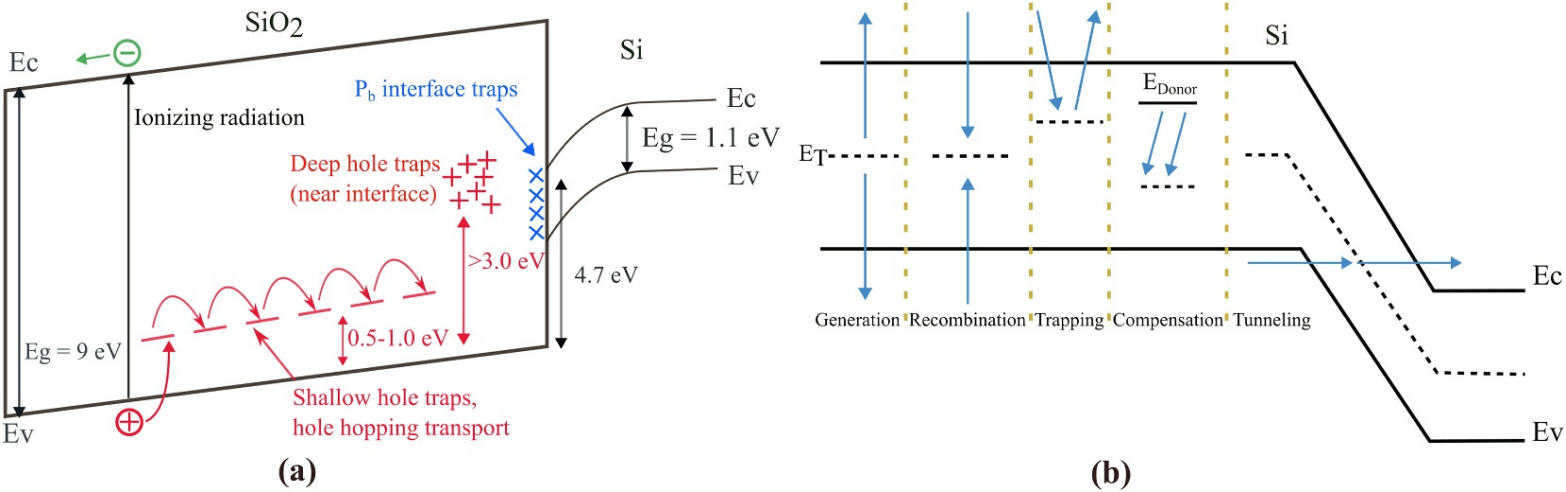
Energy‐band diagram and radiation damage mechanisms. (a) Generation, transport, and trapping of electron–hole pairs in the oxide. (b) Semiconductor energy‐band structure and defect types induced by displacement damage.

DDD effects mainly arise within the semiconductor bulk. High‐energy particles (e.g., protons, neutrons, and heavy ions) displace lattice atoms from their equilibrium positions, producing vacancies, interstitials, and more complex defect clusters, as shown in Figure 1b. Such defects introduce deep levels that act as generation–recombination centers and scattering centers, shortening carrier lifetime, enhancing nonradiative recombination, and perturbing charge transport and the internal electric‐field distribution. In photodetectors, displacement damage typically intensifies bulk generation–recombination processes, leading to a pronounced increase in dark current accompanied by elevated shot noise and generation–recombination noise. Meanwhile, the reduction in carrier lifetime and diffusion length lowers the collection efficiency of photogenerated carriers, resulting in decreased responsivity and quantum efficiency. For high‐speed photodetectors, defect‐assisted trapping/detrapping and recombination can also slow the transient response and introduce long tails, thereby degrading bandwidth and linearity and, in some cases, increasing the required operating bias or amplifying the device's sensitivity to bias conditions [37, 38].

### 2.2 Damage Simulation

For the photodetector structures used in this work, the thin‐film stack can be categorized into two representative configurations. The first is a Ge/Si heterostructure, in which an approximately 500nm germanium (Ge) absorption layer is integrated on top of a 220nm silicon (Si) functional layer. The second is a Si‐only structure, in which only the 220nm Si layer is retained without introducing a Ge absorption layer. To ensure a fair and reproducible comparison, the surrounding layers outside the active region are kept identical for both configurations: The active material is sandwiched between silicon dioxide (${\text{SiO}}_{2}$) layers of finite thickness on both the top and bottom, providing electrical isolation and surface passivation while emulating the practical cladding environment. Additionally, a silicon substrate is included at the bottom of the stack as the mechanical support and thermal/electrical reference base, yielding a multilayer material system consistent with the actual devices.

Because Ge and Si differ in density, atomic number, and stopping power, the presence or absence of the Ge absorption layer can directly modify the proton energy‐loss trajectory and the location of energy deposition within the device, thereby altering the depth‐dependent deposition profile and the deposited‐energy fractions among individual layers. Accordingly, we constructed geometric models for both configurations and performed energy‐deposition scoring and comparative analysis under identical irradiation conditions.

In the simulations, proton transport and energy deposition were modeled using the Geant4 Monte Carlo toolkit [39, 40]. The irradiation source was represented as a monoenergetic high‐energy proton beam incident normal to the surface of the multilayer structure in order to closely match the normal‐incidence irradiation geometry used in the experiments. The incident proton energy was sampled at 12 discrete points spanning 1.15–10.75MeV, which correspond one‐to‐one to the calibrated energy points of the experimental proton source. For each energy point, Monte Carlo particle transport was employed to simulate the proton energy‐loss process in the multilayer thin films, and the deposited energy was tallied in depth bins along the thickness direction to obtain the depth‐dependent energy‐deposition profile, $E\left(z\right)\equiv dE_{\text{dep}}/dz$, that is, the energy deposited per unit depth (e.g., in MeV/μm). To ensure statistical convergence and suppress fluctuations, $10^{4}$ primary proton events were accumulated and averaged at each energy. In addition, sufficiently large lateral simulation domains/world volumes were used to prevent boundary truncation from affecting secondary‐particle transport and energy‐deposition scoring. The resulting stable, energy‐resolved mean energy‐deposition distributions were then used for subsequent analysis of deposition differences among material layers and at their interfaces.

As shown in Figure 2, the depth‐dependent distribution of $dE_{\text{dep}}/dz$ indicates that the dominant energy‐deposition characteristics are primarily governed by the thick silicon region. For 1.15MeV protons, the energy loss increases progressively after entering the silicon layer, leading to a pronounced Bragg peak at approximately 18–19μm, followed by a rapid drop to nearly zero around 20μm. This suggests that the deposited energy is highly concentrated near the end of the proton range. In contrast, protons with energies of 3–11MeV exhibit a much lower deposition level within the 0–25μm depth interval, with only weak depth dependence and no evident peak, implying that their Bragg peaks occur at greater depths and that energy deposition within this thickness range is dominated by transmission. A further comparison between the Ge/Si multilayer structure and the pure‐Si structure shows that introducing the Ge/Si thin layer mainly induces local perturbations or small spikes in the vicinity of the interface at about 4–5μm, but does not alter the overall trend of the deposition profile or the location of the main peak. The dominant energy deposition still occurs deep within the subsequent silicon layer. Moreover, comparison of the deposited energy among material layers suggests that Ge yields a higher energy deposition per unit depth than Si and $SiO_{2}$ under the same irradiation conditions, whereas Si and $SiO_{2}$ exhibit comparable levels, with Si being slightly lower than $SiO_{2}$.

**FIGURE 2:**
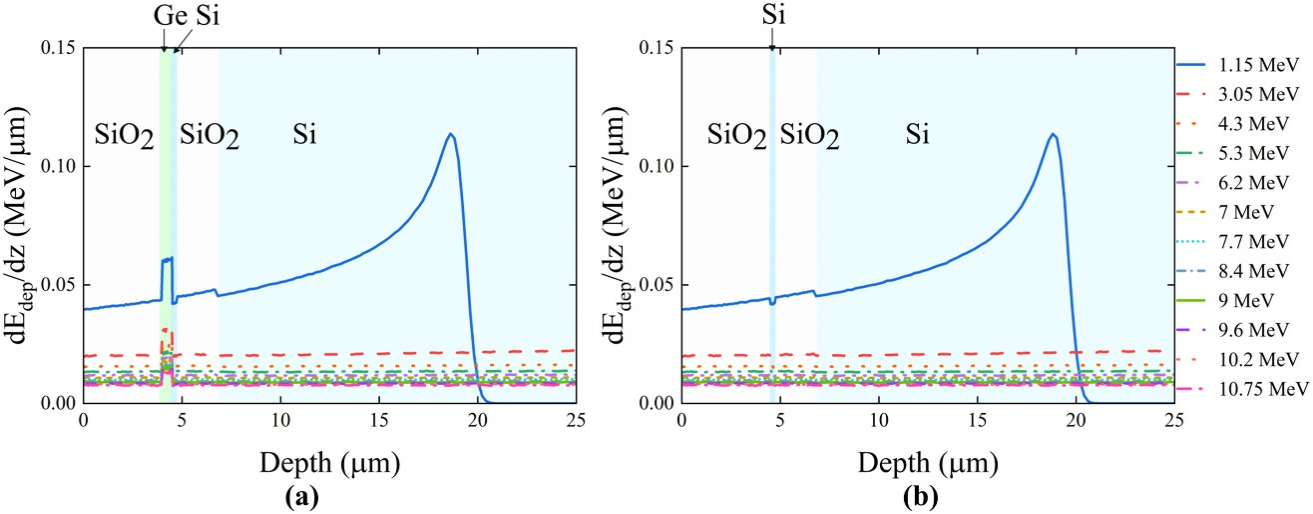
Simulation results of proton energy deposition in the detector multilayer thin‐film structure. (a) Multilayer structure with a Ge/Si stack (with an integrated Ge absorption layer). (b) Pure‐Si multilayer structure (without the Ge absorption layer) while keeping the remaining cladding and substrate stack identical.

## 3 Experiment

### 3.1 Fabrication of the Photodetector

The Ge/Si photodetectors used in the irradiation experiments were fabricated using a standard silicon‐photonic process, as shown in Figure 3a. In this layout, the detector occupies a compact footprint of $16\times 12\;\mu m^{2}$. As indicated at the bottom of the figure, the ground–signal–ground (GSG) radiofrequency electrodes are employed for electrical signal extraction. Meanwhile, an edge coupler on the right side is used to couple the optical input into the device. For clarity, the detailed device structure is further illustrated in Figure 3b. The silicon layer has a thickness of 220nm, and a 500nm‐thick germanium layer is directly epitaxially grown on top. A vertical Ge PIN junction is subsequently formed by ion implantation. In this configuration, the optical propagation direction is orthogonal to the carrier transport direction, which helps decouple optical absorption from electrical collection and supports high‐speed operation.

**FIGURE 3:**
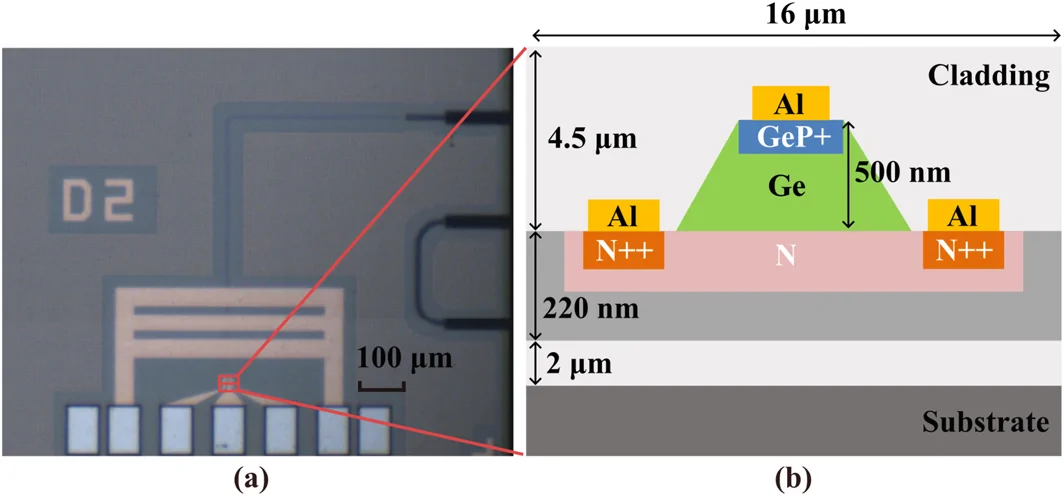
Structure of the germanium‐silicon photodetector in the irradiation experiment. (a) Micrograph of the photodetector. (b) Schematic illustration of the device structure.

### 3.2 Hydrogen Preloading Process and Proton Irradiation

During the experiments, a total of eight photodetector chips fabricated using a standard silicon‐photonic process were characterized for irradiation‐induced damage. To evaluate the passivation and repair effects of hydrogen preloading on irradiation‐induced defects, four chips were subjected to hydrogen preloading prior to irradiation. The four photodetector chips were placed in a high‐pressure reactor filled with a mixed hydrogen–nitrogen atmosphere at a temperature of 60 °C and a pressure of 4 MPa for 48 h. Under these elevated temperature and pressure conditions, hydrogen is expected to dissolve into the glassy network of silicon dioxide, where the small molecular size and chemical inertness of $H_{2}$ enable the formation of soluble and trapped storage states. Meanwhile, diffusion pathways within the ${\text{SiO}}_{2}$ matrix allow $H_{2}$ molecules to migrate and redistribute at moderate temperatures. These hydrogen species stored in the silicon dioxide act as active species participating in defect passivation once defects are generated [41, 42].

The chips were then divided into four groups, each consisting of one hydrogen‐preloaded photodetector chip and one untreated photodetector chip. The four groups of chips were arranged at fixed intervals and mounted on a sample holder. Subsequently, the sample holder was fixed with a mechanical clamp and placed inside a vacuum chamber. During proton irradiation, the photodetector chips were kept unbiased, and no optical input was applied. The samples were mounted with the device side facing the incident proton beam so that the proton beam was approximately normally incident on the chip surface. The irradiation was performed in a shot‐by‐shot manner in a vacuum. The four irradiation levels corresponded to 10, 20, 50, and 100 laser shots, respectively, with a time interval of approximately 5s between adjacent shots. Therefore, the total irradiation duration for each dose condition was mainly determined by the corresponding shot number. The broadband proton irradiation source employed in the vacuum chamber is based on a laser–plasma acceleration mechanism. When high‐intensity laser pulses interact with a solid target, an intense transient sheath field is formed at the rear surface of the target, accelerating protons to the MeV energy range within an ultrashort timescale and generating a proton beam with a broad energy spectrum [43, 44]. After this single‐stage acceleration process, the protons undergo no further acceleration or active magnetic or electric trajectory control but instead propagate in a naturally divergent manner. The irradiation area is then defined through geometric spreading and passive aperture limiting. The aperture assembly incorporates an 11‐μm‐thick aluminum (Al) foil placed upstream of the aperture to block any transmitted laser light while preserving the defined irradiation geometry, enabling controlled broadband proton irradiation of the photodetector chips. The broadband proton spectrum used here is more representative of the continuous energy distribution of space protons than a monoenergetic source and therefore provides an accelerated condition for evaluating proton‐induced degradation in spaceborne photonic devices. However, it should not be interpreted as a full reproduction of a specific orbital radiation environment because the actual proton spectrum, flux, shielding condition, and dose‐rate history depend on the mission scenario.

### 3.3 Irradiation Source Intensity Calibration

To quantify the performance degradation of the detector under proton irradiation, the device was first experimentally calibrated. The detector chip was directly exposed to a broad‐spectrum proton beam to simulate cumulative irradiation effects that may occur during long‐term operation in space. The radiation dose was calibrated using radiochromic films (RCF). The optical density (OD) obtained from scanning the films was converted to particle flux using preestablished energy‐dependent calibration curves, yielding the energy–flux spectrum at discrete energy points, covering 1.15–10.75 MeV, as shown in Figure 4a. Based on this spectrum, Monte Carlo particle transport simulations were performed to calculate the transport of the broad‐spectrum proton beam through the multilayer device structure, and the energy deposited in the silicon layer, $E_{\text{dep}}$, was recorded. The corresponding dose was then calculated according to the following equation:

(1) $$D=\frac{E_{\text{dep}}}{m_{\text{Si}}},$$

where $E_{\text{dep}}$ is the deposited energy in the silicon layer and $m_{\text{Si}}$ is the mass of the 220nm‐thick Si functional layer beneath the Ge absorption region. Here, the “dose in the silicon layer” refers to the average deposited dose in this 220nm‐thick Si functional layer rather than the entire Si substrate. This dose definition was adopted because this Si layer is the functional Si layer in the active Ge/Si photodetector structure.

**FIGURE 4:**
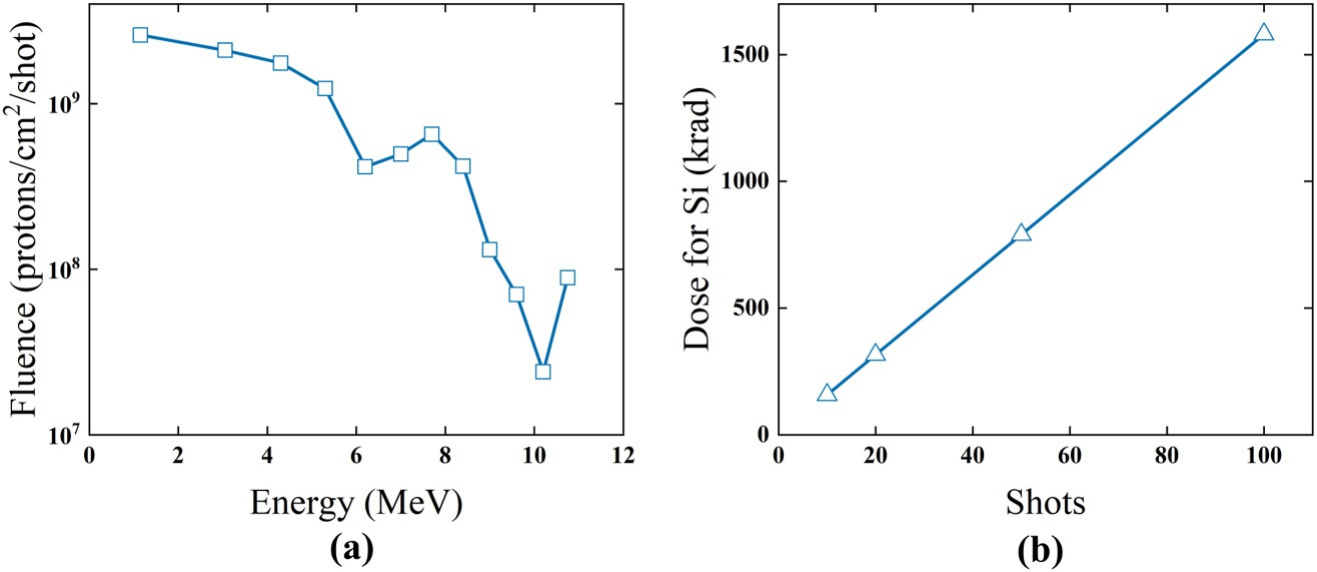
Proton energy spectrum and shot conditions used in the experiments, together with the corresponding energy‐deposition dose in the Si layer. (a) Proton energy spectrum calibrated by RCF. (b) Shot conditions and the resulting cumulative deposited dose in the Si layer.

During the irradiation experiments, four shot‐number conditions (10, 20, 50, and 100 shots) were employed to realize different proton fluences (i.e., irradiation intensities) for a systematic comparative study, thereby enabling a comprehensive evaluation of the detector performance degradation under varying irradiation environments. Based on the Monte Carlo energy‐deposition calculations described above, the deposited dose in the silicon layer per shot is 15.8krad. Consequently, the cumulative deposited doses in the silicon layer for the four shot conditions are 158, 316, 790, and 1580krad, respectively, as shown in Figure 4b.

## 4 Results and Discussion

### 4.1 Test Setup and Metrics

The measurement flow for the photodetector bandwidth is shown in Figure 5a. A combined optoelectronic frequency‐sweep method is employed. The lightwave component analyzer and performance network analyzer (LCA/PNA) generates a frequency‐swept, intensity‐modulated optical input. Prior to coupling, the input polarization is tuned with a polarization controller to maximize the coupled optical signal and detected response, after which the light is coupled into the on‐chip photodetector. Meanwhile, a fixed reverse‐bias voltage is applied to ensure operation in the high‐speed regime. The resulting RF electrical response is extracted from the chip electrodes using an RF probe and routed back to the analyzer port, where the small‐signal transfer response is recorded as the measured $S_{21}$ magnitude. To minimize the influence of the measurement chain, on‐wafer calibration is performed to shift the reference plane to the probe tips, and the measurement conditions are kept identical for all dose points to enable a consistent comparison. The amplitude–frequency response is then obtained, and the 3dB cutoff frequency, defined as the frequency at which $\left|S_{21}\right|$ drops by 3dB relative to the low‐frequency plateau, is used to quantify the bandwidth. The RF‐response measurements were performed at a wavelength of 1550nm with a reverse‐bias voltage of 2V. To ensure a consistent comparison before and after irradiation, the input optical‐power setting, optical‐coupling configuration, polarization‐adjustment procedure, and bias condition were kept unchanged throughout the measurements.

**FIGURE 5:**
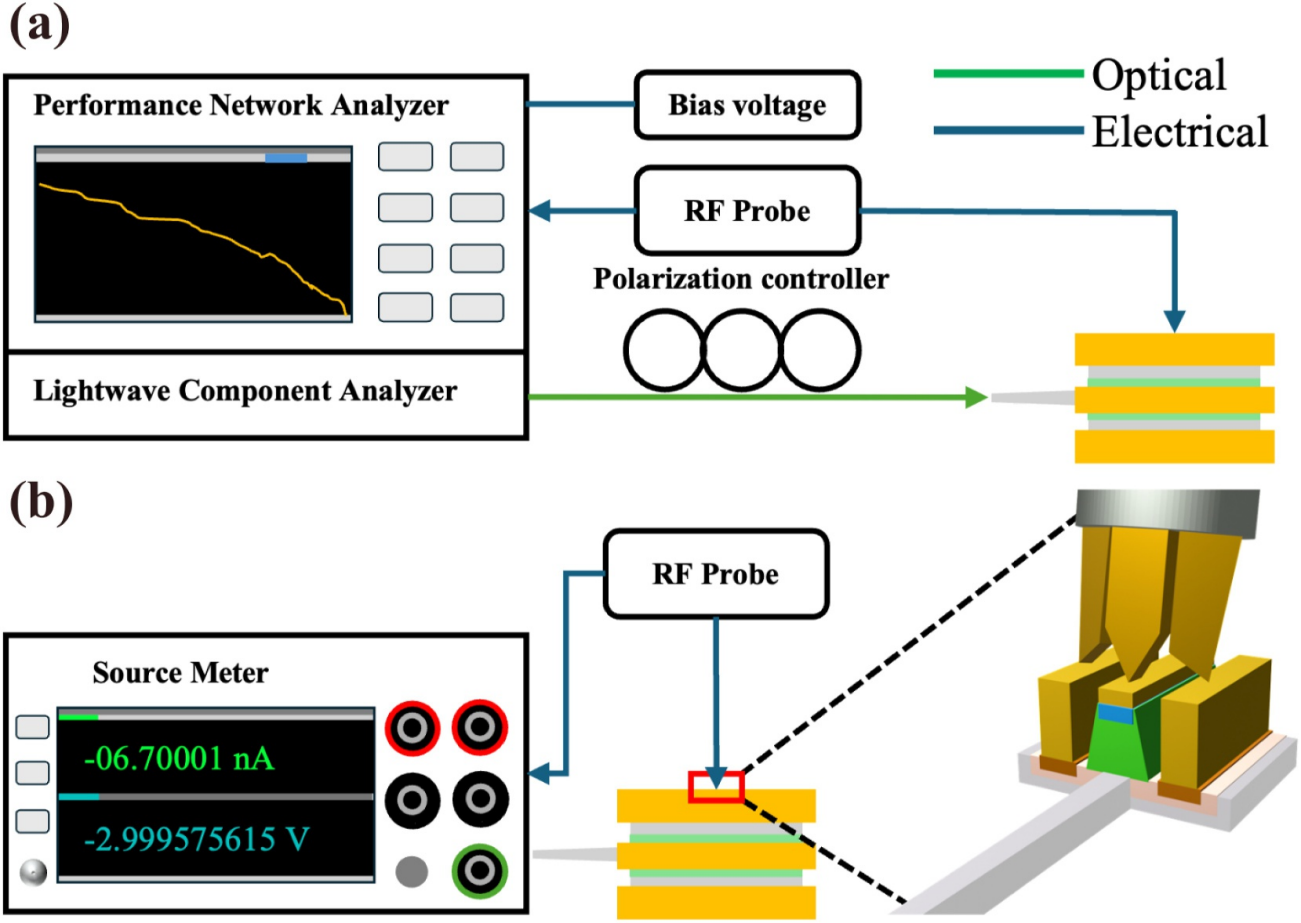
Schematic of the photodetector‐chip measurement setup. (a) Photodetector bandwidth measurement. (b) Photodetector dark‐current measurement.

In addition to the frequency‐response characterization, the dark‐current measurement flow is shown in Figure 5b. Measurements are carried out under dark conditions with no optical input, and DC electrical characterization is performed using a source meter. During the test, the source meter applies a specified reverse‐bias voltage to the photodetector through a stable probe contact to the chip electrodes. After the current reading stabilizes at each bias point, the DC current is recorded as the dark current. By sweeping the bias voltage, the dark‐current I−V characteristics are obtained, whereas an appropriate compliance limit is set to prevent device overstress. For quantitative comparison throughout this work, the dark current at a reverse bias of 3V is extracted, whereas the full I−V curves are used to evaluate irradiation‐induced changes in leakage behavior and noise‐related performance.

### 4.2 Proton Irradiation Results

Under four cumulative deposited‐dose conditions, the bandwidth and dark‐current variations of the untreated photodetectors are summarized in Figure 6. Because the dose‐dependent trends of bandwidth and dark current are highly consistent across different chips, the preirradiation performance is defined using the average of eight chips, which improves statistical representativeness and reduces the influence of chip‐to‐chip variations on the baseline reference. For the irradiated dose points in Figure 6, each curve corresponds to the untreated device assigned to the corresponding dose condition, whereas the preirradiation reference was obtained by averaging all eight tested chips.

**FIGURE 6:**
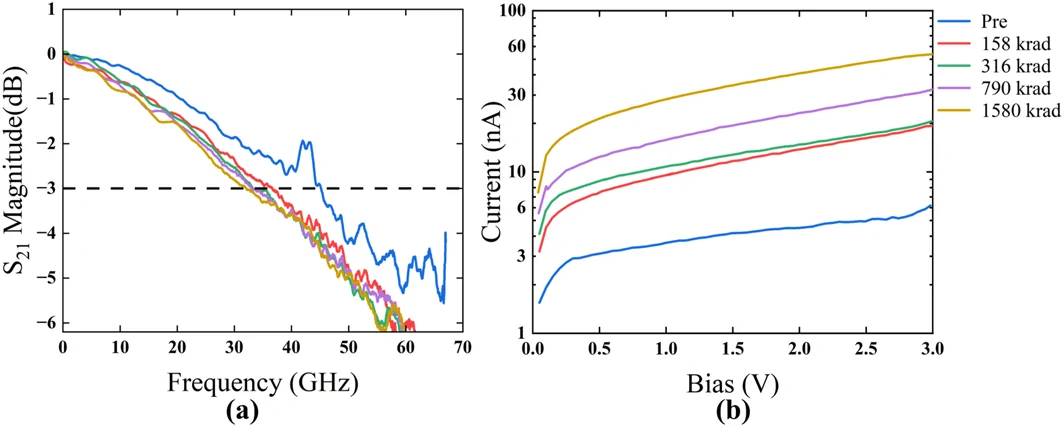
Frequency responses and dark current of untreated photodetector chips under different irradiation doses. (a) Measured $S_{21}$ under different doses. (b) Dark current under different doses.

As the proton dose increases, the device 3‐dB bandwidth exhibits a clear monotonic decrease, suggesting that irradiation‐induced defects, together with irradiation‐enhanced parasitic effects, degrade the high‐speed response. At the maximum cumulative deposited dose of 1580krad, the 3‐dB bandwidth decreases from 44.52GHz before irradiation to 32.09GHz, corresponding to 72.1% of the initial value.

Compared with the bandwidth degradation, the dark current shows a markedly stronger sensitivity to dose and increases rapidly with cumulative irradiation. At a reverse bias of 3V, the preirradiation dark current is 6.19nA and rises to 8.67× its initial value at the maximum dose. This pronounced increase in dark current implies a substantial enhancement of leakage‐related processes, which is expected to elevate noise and reduce the effective dynamic range, thereby imposing a more stringent constraint on detector performance under radiation exposure than the bandwidth reduction alone. Overall, the results in Figure 6 indicate that, for untreated devices within the investigated dose range, the irradiation‐induced degradation is manifested simultaneously in high‐speed response and leakage behavior, with the latter exhibiting a more severe dose dependence.

### 4.3 Hydrogen Loading Effects

To evaluate whether the hydrogen loading process itself introduces an initial baseline shift, the preirradiation dark‐current characteristics and normalized RF responses of untreated and hydrogen‐loaded devices were compared, as shown in Figure 7. The curves were obtained by averaging four devices in each group and were measured under the same optical‐coupling configuration, electrical biasing, and RF testing protocol, enabling a consistent comparison of the relative device baseline before irradiation.

**FIGURE 7:**
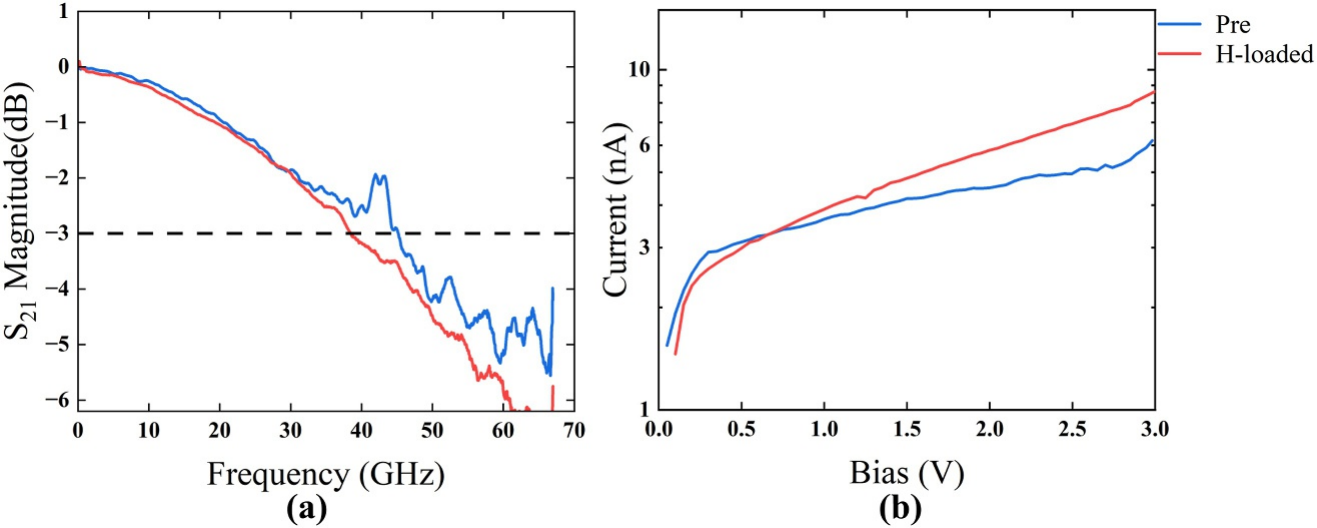
Preirradiation baseline comparison between untreated and hydrogen‐loaded Ge/Si photodetectors. (a) Averaged normalized RF responses. (b) Averaged dark‐current characteristics.

The results show that hydrogen loading may introduce a modest preirradiation penalty in certain device metrics. Specifically, the hydrogen‐loaded devices exhibit a slightly higher initial dark current, especially at larger reverse bias, and a modestly earlier RF roll‐off before irradiation. These changes may be associated with modified material and interface conditions after the high‐pressure hydrogen treatment. Nevertheless, the dark current remains at the nA level, and the devices still maintain high‐speed operation. Because the hydrogen loading treatment does not modify the junction geometry, electrode layout, pad configuration, or metallization, a large change in geometrical capacitance or pad parasitics is not expected. Therefore, the observed RF‐response shift is discussed as an effective high‐speed baseline change rather than being solely attributed to a geometrical parasitic‐capacitance variation.

From the perspective of signal‐to‐noise ratio, this limited preirradiation trade‐off is not expected to offset the radiation‐hardening benefits. The initial shift does not alter the basic operating state of the photodetector, and the devices remain fully functional under normal photoelectric detection and electrical biasing conditions. Their optoelectronic response can therefore be reliably characterized using the same measurement protocol as that used for the untreated devices.

After establishing the preirradiation baseline, the dose‐dependent characteristics of the hydrogen‐loaded devices were further investigated, as shown in Figure 8. For the irradiated dose points in Figure 8, each result corresponds to the hydrogen‐loaded device assigned to the corresponding irradiation condition. With increasing cumulative deposited dose, the hydrogen‐loaded devices still show gradual performance degradation, as expected under proton exposure. Importantly, however, both the rate of degradation and the overall magnitude of deterioration are substantially reduced compared with those of the untreated devices, indicating improved irradiation stability and resilience.

**FIGURE 8:**
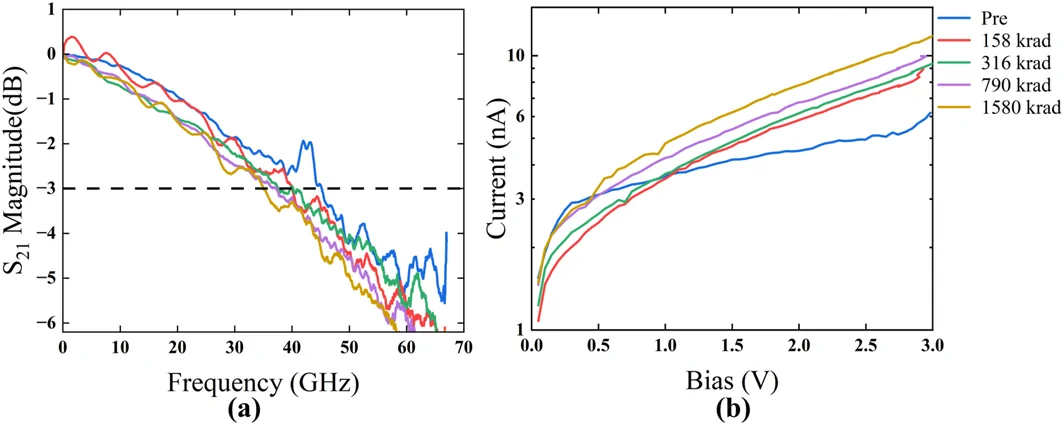
Frequency responses and dark current of hydrogen‐loaded photodetector chips under different irradiation doses. (a) Measured $S_{21}$ under different doses. (b) Dark current under different doses.

At the maximum cumulative deposited dose, the 3‐dB bandwidth of the hydrogen‐loaded devices remains at approximately 35GHz, corresponding to 78.6% of the preirradiation reference, and thus retains a higher high‐speed operating margin under the harshest irradiation condition. In parallel, at a reverse bias of 3V, the dark current increases only to 1.9× its preirradiation value, demonstrating pronounced suppression of irradiation‐induced leakage growth. Because dark current directly impacts shot noise and generation–recombination noise, such suppression is expected to reduce noise‐related degradation and help preserve the usable dynamic range of the detector.

Taken together, the results confirm that hydrogen loading provides a robust hardening benefit for integrated photodetectors. Although hydrogen loading introduces a limited preirradiation baseline shift, this modest trade‐off is not expected to outweigh the subsequent radiation‐hardening benefit, particularly considering the suppressed dark‐current degradation and improved bandwidth retention after proton irradiation. The most significant improvement is observed in the mitigation of dark‐current amplification, whereas bandwidth degradation is also alleviated. This overall behavior is consistent with hydrogen‐assisted passivation and electrical deactivation of irradiation‐related defect states, which can reduce defect‐mediated generation–recombination processes and limit the activation of additional leakage pathways during proton irradiation.

### 4.4 Discussion on Degradation and Hardening Mechanisms

As discussed above, radiation‐induced degradation in photodetectors can be broadly categorized into TID and DDD, as schematically summarized in Figure 9a. TID is mainly associated with defect generation and activation in dielectric layers and at oxide/semiconductor interfaces, whereas DDD originates from elastic collisions between energetic protons and lattice atoms in the semiconductor, producing vacancies, interstitials, and their complexes (Frenkel pairs), as illustrated for both Ge and Si regions in Figure 9a. In this work, the bias‐dependent dark‐current characteristics indicate that hydrogen loading suppresses the irradiation‐induced leakage increase over the measured reverse‐bias range. This behavior is consistent with a reduction in electrically active defect‐assisted leakage pathways. However, the present measurements cannot uniquely separate the contributions of oxide/interface‐related defects from bulk displacement‐damage‐related defects. Therefore, the hydrogen‐assisted hardening effect is discussed here in terms of passivation or electrical deactivation of electrically active defect states rather than as direct physical repair of displacement damage or as an exclusively interface‐limited mechanism. For Ge/Si integrated photodetectors, the radiation‐induced defect landscape can be more complex than the representative oxide $E^{\prime }$‐center and Si/$SiO_{2}\;P_{b}$‐center models. In addition to oxide and Si/$SiO_{2}$‐related defects, Ge bulk defects, Ge/Si heterointerface states, Ge/oxide interface traps, and irradiation‐induced recombination centers in the depletion or near‐junction regions may also contribute to carrier trapping, nonradiative recombination, and leakage‐current enhancement. Therefore, the hydrogen loading effect should be understood as the partial passivation or electrical deactivation of accessible electrically active defects rather than as a mechanism limited only to Si/$SiO_{2}$ interface passivation.

**FIGURE 9:**
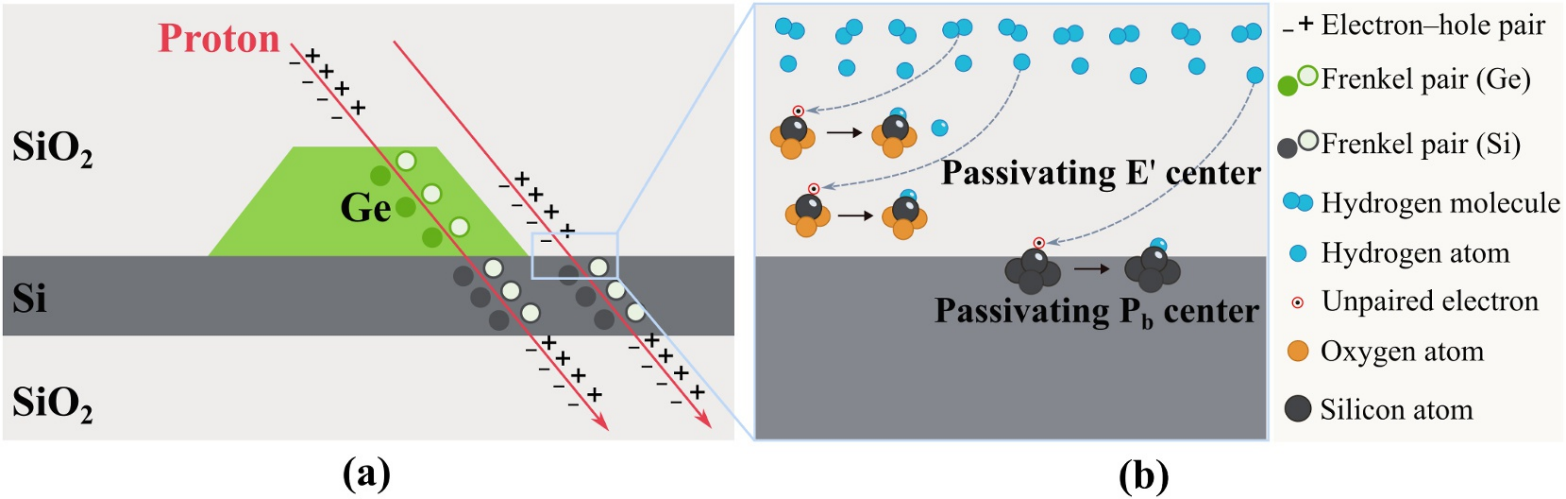
Schematic illustration of proton‐irradiation‐induced defect generation and hydrogen passivation mechanisms in Ge/Si integrated photodetectors. (a) Under proton irradiation, ionizing interactions in the $SiO_{2}$ layers generate electron–hole pairs, while nonionizing collisions in the Ge and Si regions create displacement damage in the form of Frenkel pairs. (b) Hydrogen stored in $SiO_{2}$ as $H_{2}$ can supply reactive atomic hydrogen $\left(H^{0}\right)$, which passivates dangling‐bond‐type defects by forming Si–H bonds, including oxide $E^{\prime }$ centers and interfacial $P_{b}$ centers.

For TID, ionizing radiation generates electron–hole pairs in the $SiO_{2}$ network. Owing to their high mobility, electrons are rapidly swept out or recombine, whereas holes drift under the electric field and become trapped. Such trapping events can promote local bond breaking and structural rearrangement of the Si−O network, thereby creating electrically active oxide defects characterized by Si dangling bonds. A representative oxide defect is the $E^{\prime }$ center, which is commonly described by an oxygen‐vacancy‐related, threefold‐coordinated Si dangling‐bond configuration, denoted as $O_{3}\equiv Si•$, as depicted in Figure 9b. This structure can be viewed as a defect configuration formed after the rupture and reconstruction of local ≡Si−O−Si≡ bonding environments during irradiation and hole trapping.

In addition to bulk‐oxide defects, irradiation can also induce or enhance interface dangling‐bond defects at the oxide/semiconductor interface. For the Si/$SiO_{2}$ interface, the $P_{b}$ center is typically represented as an interfacial Si dangling bond, ≡Si•, which is closely related to the depassivation of hydrogen‐terminated bonds, as schematically indicated in Figure 9b. This depassivation process can be described in terms of bond chemistry as follows:

(2) $$\equiv \;Si-H\to \equiv Si•+H^{0},$$

where $H^{0}$ denotes neutral atomic hydrogen. Notably, the term “$P_{b}$ center” is conventionally used for dangling‐bond defects at the Si/$SiO_{2}$ interface. In the Ge/Si heterostructure studied here, other interfaces (e.g., Ge/oxide) may exhibit analogous dangling‐bond/trap states. However, we adopt the Si/$SiO_{2}\;P_{b}$‐center picture as a representative model for interface‐related leakage and hydrogen passivation without explicitly distinguishing the contributions from different semiconductor/oxide interfaces. Structurally, both $E^{\prime }$ centers in the oxide and $P_{b}$ centers at the interface correspond to unsatisfied Si dangling bonds. These defect sites provide active pathways for defect‐assisted generation–recombination and leakage processes under reverse bias, thereby leading to pronounced increases in dark current and noise.

Hydrogen loading offers a direct chemical passivation route for dangling‐bond‐type defects, as summarized in Figure 9b. In $SiO_{2}$, hydrogen is predominantly stored as molecular hydrogen $H_{2}$, serving as a stable reservoir. Under irradiation and typical device operating conditions, $H_{2}$ can undergo dissociation or hydrogen‐transfer reactions in the vicinity of defect sites, producing reactive neutral atomic hydrogen $H^{0}$. The resulting $H^{0}$ terminates dangling bonds and forms Si–H bonds, thereby deactivating electrically active defect sites [45, 46, 47, 48]. The passivation reactions can be written as follows:

(3) $$\begin{array}{cc} \equiv \;Si•+H^{0} & \to \equiv Si-H,\\ O_{3}\;\equiv \;Si•+H^{0} & \to O_{3}\equiv Si-H,\\ O_{3}\;\equiv \;Si•+H_{2} & \to O_{3}\equiv Si-H+H^{0}. \end{array}$$

The long‐term stability of the hydrogen‐assisted passivation effect is also an important reliability issue for spaceborne applications. Although the formation of Si–H bonds can electrically deactivate dangling‐bond‐type defects, the retention of hydrogen‐related passivation may be affected by long‐term aging, radiation‐enhanced diffusion, hydrogen redistribution, out‐diffusion, or depassivation during repeated thermal cycling. In particular, severe temperature variations in space environments may weaken the passivation effect over extended mission durations by promoting hydrogen migration or reactivation of previously passivated defect sites. Therefore, the present results demonstrate the effectiveness of hydrogen loading under the investigated proton‐irradiation and measurement conditions, but they should not be interpreted as a complete validation of multiyear passivation stability. Long‐term thermal‐aging, thermal‐cycling, and postirradiation retention experiments will be required in future work to further assess the durability of the hydrogen‐assisted hardening effect under realistic space‐mission conditions.

By contrast, DDD is driven by nonionizing energy loss in the semiconductor and is commonly described by the creation of point defects, such as vacancies (V) and interstitials (I), and their subsequent reactions into more stable complexes or clusters, which can be schematically represented as follows:

(4) $$Si\overset{displacement}{\to }V+I.$$

These displacement‐related defects introduce broken or strained bonds in the lattice and can act as electrically active centers that enhance bulk generation–recombination processes and contribute to leakage, consistent with the Frenkel‐pair picture illustrated in Figure 9a. Although $H^{0}$ cannot repair the displaced lattice or eliminate the primary displacement damage, it may reduce the electrical activity of certain defect states that are accessible to hydrogen, particularly at oxide/semiconductor interfaces, sidewalls, near‐surface regions, and near‐junction leakage paths. Therefore, the observed dark‐current suppression is more cautiously attributed to hydrogen‐assisted electrical deactivation of electrically active leakage‐related defect states rather than to direct repair of lattice displacement or to an exclusively interface‐dominated passivation mechanism.

To facilitate a side‐by‐side comparison between hydrogen‐loaded and untreated devices under identical dose conditions, the results are consolidated in Figure 10. The bandwidth metrics are summarized in Figure 10a, where both the absolute 3‐dB bandwidth and the normalized values referenced to the preirradiation baseline are presented. The corresponding dark‐current characteristics are compiled in Figure 10b, including the absolute current at a 3V reverse bias and the change factor relative to the preirradiation reference. This unified presentation highlights that hydrogen loading simultaneously mitigates bandwidth degradation and suppresses irradiation‐induced leakage growth over the entire dose range.

**FIGURE 10:**
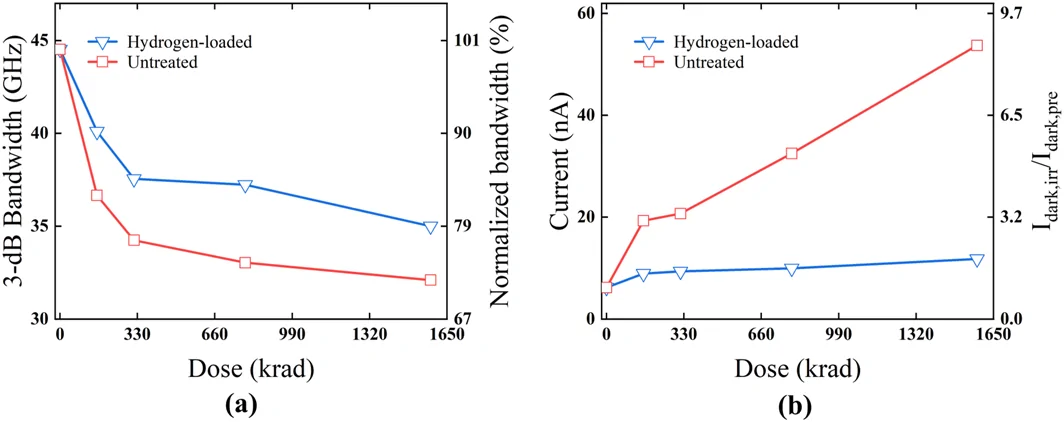
Dose‐dependent 3‐dB bandwidth and dark current measured at a reverse bias of 3V. (a) 3‐dB bandwidth versus irradiation dose. (b) Dark current versus irradiation dose. The irradiated data points are single‐device results at each dose condition, and the preirradiation reference is the averaged baseline.

The bandwidth degradation can be further interpreted using a simplified small‐signal equivalent‐circuit framework. The Ge/Si photodetector can be approximately represented by a photocurrent source $I_{\text{ph}}$ in parallel with the junction capacitance $C_{j}$, shunt/leakage resistance $R_{\text{sh}}$, and parasitic capacitance $C_{p}$, followed by a series resistance $R_{s}$ and the external 50Ω readout load $R_{L}$. The effective resistance seen by the junction and parasitic capacitances can be written as $R_{\text{eff}}\approx R_{\text{sh}}\|\left(R_{s}+R_{L}\right)$. When $R_{\text{sh}}$ is much larger than the external loading path, the RC‐limited bandwidth can be approximated as follows:

(5) $$f_{\text{RC}}\approx \frac{1}{2\pi \left(R_{s}+R_{L}\right)\left(C_{j}+C_{p}\right)}.$$

The measured 3‐dB bandwidth is also influenced by carrier transport and can be phenomenologically described as follows:

(6) $$\frac{1}{f_{3dB}^{2}}\approx \frac{1}{f_{\text{tr}}^{2}}+\frac{1}{f_{\text{RC}}^{2}}.$$

Accordingly, proton irradiation may degrade the bandwidth through several coupled pathways. Trapped charges and radiation‐induced defect states can modify the depletion profile and local electric‐field distribution, resulting in an effective change in $C_{j}$ or $C_{p}$. Meanwhile, displacement‐related defects, mobility degradation, trap‐assisted transport, or contact/doped‐region degradation may increase the effective $R_{s}$. Radiation‐induced trapping, detrapping, and recombination can also slow the carrier‐transit process and reduce $f_{\text{tr}}$.

The experimental trends suggest that leakage‐related degradation is more sensitive to proton irradiation than the high‐speed response within the investigated dose range. For untreated devices, the bandwidth decreases from 44.52GHz to 32.09GHz, whereas the dark current increases to 8.67× its preirradiation value. In contrast, hydrogen‐loaded devices retain approximately 35GHz bandwidth while limiting the dark‐current increase to 1.9×. This comparison suggests that hydrogen loading primarily suppresses electrically active leakage‐related defect pathways, whereas the improvement in bandwidth retention may arise from reduced trap charging/discharging, weaker local‐field perturbation, and moderated effective RC/transport degradation.

The different sensitivities of bandwidth and dark current to proton irradiation can therefore be attributed to their distinct governing mechanisms. Within the investigated dose range, the high‐speed response is affected by effective RC and carrier transport changes, but the degradation remains moderate and the devices continue to operate in a high‐speed regime. By contrast, dark current is dominated by generation–recombination processes in the junction and at surface or interface regions. Proton‐induced displacement damage and interface‐related traps introduce deep‐level centers that enable additional generation–recombination pathways and can activate trap‐assisted tunneling or surface leakage channels, leading to a rapid increase in dark current. This effect can be further amplified in compact integrated devices where surface states and edge fields play a more pronounced role.

Responsivity and external quantum efficiency are also essential metrics for spaceborne photonic links because they are directly related to the carrier collection efficiency of the photodetector. Radiation‐induced defects can introduce nonradiative recombination centers and carrier traps in the Ge/Si junction and near‐interface regions, which may reduce the probability that photogenerated carriers are collected before recombination. Therefore, bandwidth retention alone should not be interpreted as complete preservation of photodetector performance. In this work, the observed suppression of dark‐current increase in hydrogen‐loaded devices indicates that hydrogen loading reduces the electrical activity of leakage‐related and recombination‐related defect pathways. This effect is beneficial for maintaining a lower noise floor and may also mitigate defect‐assisted carrier loss, although the present measurements mainly quantify leakage/noise‐related degradation and high‐speed‐response degradation rather than absolute optical responsivity.

Overall, hydrogen loading should be regarded as a mitigation strategy that reduces the electrical activity of leakage‐related defect states rather than a direct repair of displacement damage or a complete validation of long‐term space reliability. The most evident benefit observed in this work is the suppression of irradiation‐induced dark‐current growth, whereas the improved bandwidth retention can be understood as moderated effective RC/transport degradation. Although hydrogen loading introduces a limited preirradiation baseline shift, the devices remain fully functional, and the postirradiation hardening benefit is substantial under the investigated conditions.

## 5 Conclusion

In summary, we implemented a dose‐calibrated workflow for broad‐spectrum proton irradiation of integrated photodetectors by combining RCF spectrum calibration with Geant4 energy‐deposition simulations and evaluated device performance at cumulative deposited doses of 158, 316, 790, and 1580krad in the silicon layer. For untreated devices, the bandwidth decreases moderately from 44.52GHz to 32.09GHz (72.1%) at the maximum dose, whereas the dark current at a 3V reverse bias increases sharply to 8.67× its preirradiation level, highlighting leakage/noise degradation as the dominant reliability concern. A hydrogen preloading hardening process markedly improves irradiation tolerance, with devices remaining functional while preserving ∼35GHz bandwidth (78.6%) and limiting the dark‐current increase to 1.9× at 1580krad. These results offer a dose‐traceable experimental basis and a quantitative methodology to support radiation‐hardening process development and long‐term reliability optimization of integrated photodetectors for space‐relevant applications.

## Author Contributions

**Junguo Xu:** software, methodology, conceptualization, investigation, validation, writing – review and editing, writing – original draft, data curation. **Yingying Zhong:** methodology, validation, investigation, data curation. **Xiongping Bao:** methodology, software. **Junfeng Chen:** methodology, software. **Wenjie Peng:** methodology. **Haotian Hu:** methodology. **Yiti Xiong:** data curation, methodology. **Weiye Xia:** methodology, data curation. **Xiao Chen:** methodology. **Meisong Liao:** methodology. **Jianhui Bin:** methodology, resources. **Yuchen Wang:** resources, writing – review and editing, methodology. **Libing Zhou:** funding acquisition, resources, project administration, methodology, supervision, writing – review and editing. **Weibiao Chen:** methodology.

## Conflicts of Interest

The authors declare no conflicts of interest.

## Data Availability

The data that support the findings of this study are available within the article.
